# Analgesic Effect of Indian Gooseberry (*Emblica officinalis* Fruit) Extracts on Postoperative and Neuropathic Pain in Rats

**DOI:** 10.3390/nu8120760

**Published:** 2016-11-26

**Authors:** Dong Wook Lim, Jae Goo Kim, Yun Tai Kim

**Affiliations:** 1 Research Group of Innovative Special Food, Korea Food Research Institute, 62, Anyangpangyo-ro, Bundang-gu, Seongnam 13539, Korea; neodw4015@kfri.re.kr (D.W.L.); Kim.Jae-goo@kfri.re.kr (J.G.K.); 2 Department of Food Biotechnology, Korea University of Science & Technology, 217 Gajeong-ro, Yuseong-gu, Daejeon 34113, Korea

**Keywords:** analgesic effects, anti-inflammation, *Emblica officinalis*, Indian gooseberry, pain, plantar incision, spared nerve injury

## Abstract

Indian gooseberry (*Emblica officinalis* fruit), also known as “Amla” is one of the oldest edible fruits known in India. It has also traditionally been used to treat inflammation, and as an analgesic to treat wounds. However, experimental evidence for the analgesic effects of *E. officinalis* has been lacking. The present study investigated whether *E. officinalis* extracts exhibit analgesic effects in the plantar incision (PI) and spared nerve injury (SNI) pain-model rats. We evaluated the mechanical withdrawal threshold (MWT) using von Frey filaments, and pain-related behavior was determined after surgery based on ultrasonic vocalization (USV). The group treated with *E. officinalis* extracts at 300 mg/kg had significantly increased MWT values at 6 h and 24 h after the PI, and had a significantly reduced number of 22–27-kHz USVs at 6 h and 24 h after PI. Moreover, after 15 days of continuous treatment with *E*. *officinalis* extracts, the treated group showed significantly alleviated SNI-induced hypersensitivity and reduced pro-inflammatory cytokine levels. Thus, *E. officinalis* extracts have potential analgesic effects in both postoperative and neuropathic pain models in vivo.

## 1. Introduction

The therapeutic agents currently available today for the treatment of pain usually have limited effectiveness and safety [1]. The repeated use of non-steroidal anti-inflammatory drugs (NSAIDs) may induce several adverse effects, such as gastrointestinal lesions or renal and liver failure [2]. In addition, the use of currently available analgesic agents, including opioids, is often hampered by undesired dose-limiting side effects, such as tolerance and physical dependence [3]. Therefore, research into new effective and safe analgesic agents with satisfactory tolerability and proven efficacy is urgently needed [4]. Recently, alternative agents, such as natural products, have been shown to contain richly diverse compounds, leading to discovery of compounds with medical applications, particularly in the treatment of pain [5,6,7].

Indian gooseberry (the fruits of *Emblica officinalis*) commonly known as ”Amla” is one of the oldest known edible fruits in India. It belongs to the family Euphorbeaceae and is native to India, Sri Lanka, Malaysia, and China [8]. The fruits are used extensively in Ayurveda medicines as a potent ”Rasayana” group of drugs, as a health-promoting and disease-preventive tonic, and as a major component of the formulation known as ”Chyawanprash”, which is one of the most popular Ayurvedic preparations [9,10].

Amla is highly nutritious and is an important dietary source of vitamin C, minerals, and amino acids. It is also contains phenolic compounds, tannins, phyllembelic acid, phyllemblin, rutin, curcuminoides, and emblicol [11]. *E. officinalis* extracts have also been reported to possess hypolipidemic [12,13], anti-obesity [14], anti-diabetic [15], anti-cancer [16], hepatoprotective [17], and anti-inflammatory [18] activities. A pilot clinical study showed a reduction in the total and LDL cholesterol levels, enhancement of beneficial HDL cholesterol levels, and reduction in blood levels of CRP levels, a marker of inflammation, in response to Amla supplements [19]. However, while *E. officinalis* extracts have been reported to exert anti-nociceptive or analgesic activities in various animal models [20,21], little is known about the analgesic effects of *E. officinalis* extracts on surgical incision postoperative pain or neuropathic pain in vivo models.

The present study investigated whether *E. officinalis* extracts exhibit analgesic effects in a model of postoperative pain generated through plantar incision (PI) [22] and on the spared nerve injury (SNI) rat model of neuropathic pain [23]. To evaluate pain-related behavior, we studied the mechanical withdrawal threshold (MWT), as measured by von Frey filaments, and examined pain-induced ultrasonic vocalization (USV) by using ultrasonic microphones [24]. In addition, pain-related cytokine levels were determined in the dorsal root ganglia (DRG) of the SNI model rats.

## 2. Materials and Methods

### 2.1. Preparation of E. officinalis Extracts

*E. officinalis* was purchased from the Himalaya Drug Company (Makali, Bangalore, India). The voucher specimen was deposited in the Research Group of Innovative Special Food, Korea Food Research Institute. *E. officinalis* (300 g) was extracted with 70% ethanol (3000 mL) for 4 h at 80 °C in a reflux apparatus. The process was repeated once, and the extracts were combined and filtered through a membrane filter (0.45 µm; Millipore, Billerica, MA, USA). The samples were lyophilized to yield a dark yellow powder. The yield of *E. officinalis* extracts was 18.8%.

### 2.2. Animals and Treatments

All animal experiments were carried out according to the guidelines of the Korea Food Research Institutional Animal Care and Use Committee (KFRI-M-13003-1).

Male Sprague-Dawley (SD) rats (Samtako Bio Korea, Gyeonggi-do, Korea) weighing 180–210 g were housed at two rats per cage under a controlled temperature (23 ± 1 °C) and a 12 h light/dark cycle (lights on at 07:00 and lights off at 19:00). The rats were allowed at least 1 week for acclimatization before the experiments, and they were anesthetized with 2% of isoflurane prior to surgery. 

After the PI surgery, rats were divided into the following three treatment groups: (1) PI + vehicle; (2) PI + naproxen (30 mg/kg); and (3) PI + *E. officinalis* extracts (300 mg/kg). *E. officinalis* extracts were dissolved in distilled water for oral administration at the desired doses, in a volume of 5 mL/kg. *E. officinalis* extracts were administered orally, immediately after the plantar incision operation. 

After the SNI operation, rats were divided into the following three treatment groups: (1) SNI + vehicle; (2) SNI + naproxen (30 mg/kg); and (3) SNI + *E. officinalis* extracts (300 mg/kg). *E. officinalis* extracts were also administered orally, immediately after surgery, once a day, continued for 15 consecutive days. Naproxen (Sigma-Aldrich Co., St. Louis, MO, USA) was dissolved in 0.9% saline solution, and it was injected intraperitoneally (i.p., an injection volume of 3 mL/kg). All animal experiments were carried out according to the guidelines of the Korea Food Research Institutional Animal Care and Use Committee (KFRI-M-13003-1).

### 2.3. Plantar Incision of Postoperative Pain Rat Model

PI Surgery was performed as previously described [22], with minor modifications. Briefly, rats were anaesthetized with 2% isofluorane, and a 1 cm longitudinal incision was made with a scalpel, through the skin and fascia of the plantar aspect of the paw, starting 0.5 cm from the proximal edge of the heel and extending toward the toes. The plantar is muscle was elevated and incised longitudinally. Following hemostasis via gentle pressure, the skin was opposed with two single interrupted sutures using polyamide monofilaments. The animals were allowed recovery in their home cages.

### 2.4. Ultrasonic Vocalization Analysis

Pain-induced USV measurement was carried out in rats as previously described [25]. After induction of PI-related postoperative pain, USVs at 22–27 kHz emitted by the adult rats was monitored and scored for 10 min, using Sonotrack ultrasonic microphones (Metris B.V., KA Hoofddorp, The Netherlands) placed at a distance of 25–30 cm from the heads of the animals. The rats emitted ”calls” that were counted using Sonotrack 2.2.1 software (Metris, Hoofddorp, The Netherlands).

### 2.5. Spared Nerve Injury of the Neuropathic Pain Rat Model

Surgery was performed as previously described [26], with minor modifications. The SNI procedure comprised an axotomy and ligation of the tibial and common peroneal nerves leaving the sural nerve intact. The common peroneal and the tibial nerves were tight-ligated with 5.0 silk and sectioned distal to the ligation, removing 2 ± 4 mm of the distal nerve stump. Great care was taken to avoid any contact with or stretching of the intact sural nerve. The skin was opposed with two single interrupted sutures using polyamide monofilaments. In sham controls, the sciatic nerve and its branches were identically exposed, but were neither ligated nor transected.

### 2.6. Mechanical Withdrawal Threshold Analysis

Animals were placed on an elevated wire grid, and the plantar surface of the paw stimulated with a series of ascending force von Frey monofilaments (Stoelting, Wood Dale, IL, USA). The threshold was taken as the lowest force that evoked a brisk withdrawal response to one of three repetitive stimuli. To determine the time course of hyperalgesia, a baseline measurement was made prior to surgery, and then again at 6 h and 24 h post-surgery for plantar incision, 3, 6, 9, 12, and 15 days post-surgery for SNI.

### 2.7. Cytokine Analysis

The measurements of interleukin-1β (IL-1β), IL-2, IL-6, IL-12, and IL-10 levels in the isolated L4, L5, and L6 dorsal root ganglia of SNI-injured rats were performed according to the manufacturer’s instructions by using the multiplex ELISA cytokine assays (Quansys Biosciences, Logan, UT, USA, BioLegend, San Diego, CA, USA).

### 2.8. Statistical Analysis

Data analyses were performed using one-way analysis of variance (ANOVA), followed by Tukey’s post hoc test, using Prism 5 (GraphPad Software, Inc., San Diego, CA, USA) for multigroup comparisons. All data were presented as the mean ± standard error mean (SEM). Significance was set at *p* < 0.05.

## 3. Results

### 3.1. Effects of E. officinalis Extracts on Mechanical Hyperalgesia Induced by Plantar Incision

**Figure 1:**
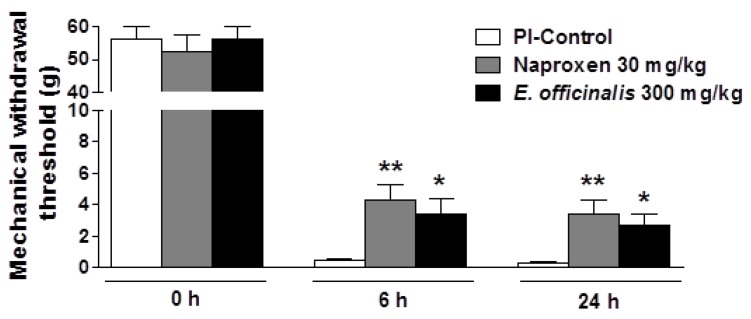
Effect of *Emblica officinalis* extracts on mechanical hypersensitivity induced by plantar incision in rats. Baseline assessment of animals before surgery (day 0) showed no significant variation between groups. At 6 h or 24 h after PI, rats treated with *E. officinalis* extracts demonstrated significantly attenuated hypersensitivity in response to von Frey stimulation of the injured hind paw. Data are mean ± SEM (*n* = 9 per group). * *p* < 0.05 and ** *p* < 0.01, significant difference from the control group. PI-Control: plantar incision in control group.

Incision of the plantar surface of the hind paw produced a significant reduction in the MWT, as measured using the von Frey assays. PI produced a marked mechanical hyperalgesia in the incised paw (MWT reduced from 56.22 ± 3.78 g at baseline to 0.32 ± 0.06 g 24 h after PI). The oral administration of *E. officinalis* extracts (300 mg/kg) and naproxen (as a positive control) significantly attenuated mechanical hyperalgesia in response to von Frey stimulation of the injured hind paw, as evidenced by increased MWT values as compared to control rats (Figure 1), and were not significantly different between *E. officinalis* extract- and naproxen-treated groups.

### 3.2. Effects of E. officinalis Extracts on USV Induced by Plantar Incision

**Figure 2:**
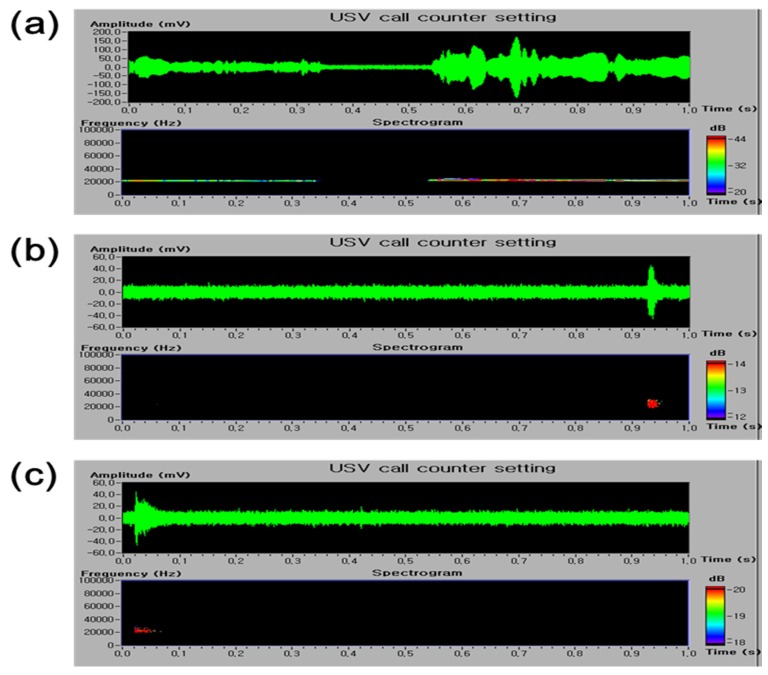

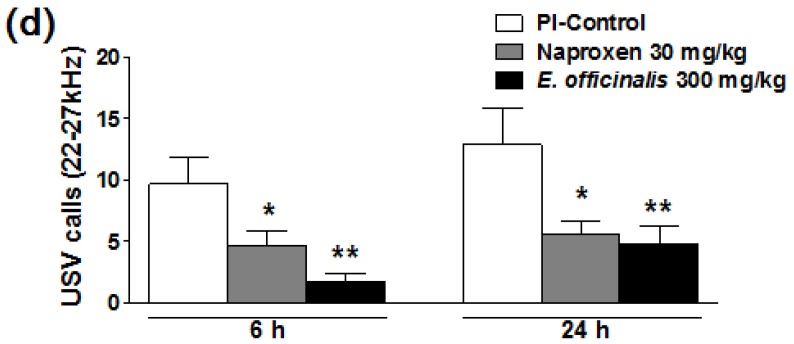
Effect of *E. officinalis* extracts on ultrasonic vocalization (USV) induced by plantar incision in rats. The sonograms of USVs in a (**a**) control; (**b**) naproxen; and (**c**) *E. officinalis* extracts-treated rats; (**d**) A significant difference was observed in the number of 22–27 kHz USV calls was observed between the *E. officinalis* extract-treated group and the control group. Data represent the mean ± SEM (*n* = 9 per group). * *p* < 0.05 and ** *p* < 0.01, significant difference from the control group. PI-Control: plantar incision in the control group.

The analgesic activity of *E. officinalis* extracts was also confirmed by pain-induced USV using ultrasonic microphones. After 6 h or 24 h after PI, the control group emitted 22–27 kHz USV calls, which is considered a pain-related behavior. However, the naproxen-treated group showed significantly reduced 22–27 kHz USV calls. Additionally, *E. officinalis* extracts reduced the number of 22–27 kHz USVs; a significant reduction was observed after the administration of *E. officinalis* extracts at a dose of 300 mg/kg (Figure 2). The number of 22–27 kHz calls were not significantly different between the *E. officinalis* extract- and naproxen-treated groups.

### 3.3. Effects of E. officinalis Extracts on Mechanical Hyperalgesia Induced by SNI

**Figure 3:**
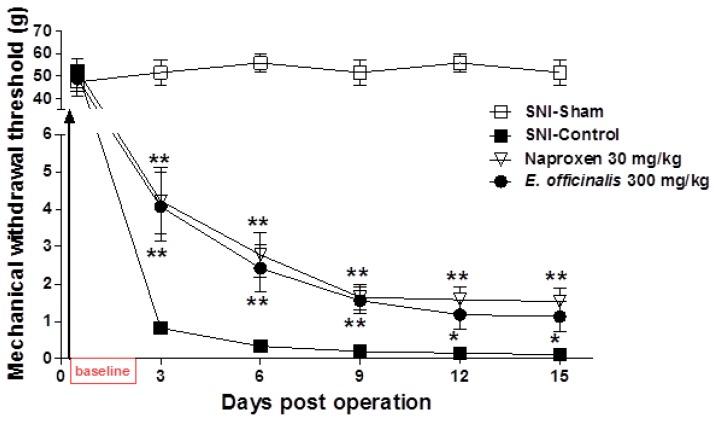
Effect of *E. officinalis* extracts on a spared nerve injury (SNI) rat model of neuropathic pain. Administration of *E. officinalis* extracts (300 mg/kg, oral administration (p.o.)) significantly attenuated hypersensitivity in response to von Frey stimulation of the hind paw, from 3 to 15 days after treatment. Data represent the mean ± SEM (*n* = 9 per group). ** *p* < 0.01, and * *p* < 0.05 significant difference from the control group.

In this study, we evaluated the potential efficacy of systemic administration of *E. officinalis* extracts in a SNI rat model of neuropathic pain. At baseline (day 0), no significant changes were observed between the group that was treated with *E. officinalis* extracts and the control group. Three days post operation, animals began to show a hypersensitivity response to von Frey stimulation, which lasted throughout the study. However, administration of *E. officinalis* extracts (300 mg/kg) significantly attenuated hyperalgesia in response to von Frey stimulation of the hind paw, as evidenced by an increased MWT as compared to SNI-control rats, from 3 to 15 days after treatment (Figure 3).

### 3.4. Effects of E. officinalis Extracts on the Expression of Cytokines Induced by SNI in Rat DRG

The IL-1β, IL-2, IL-6, and IL-12 levels were measured in the isolated L4, L5, and L6 DRG of the rats, and their levels were significantly increased in the SNI control group. Moreover, the IL-10 level was significantly decreased in the SNI-control group compared with the sham group. The SNI + *E. officinalis* extract-treated-group (300 mg/kg) and the SNI + naproxen treated group (30 mg/kg) had showed significantly lower IL-1β, IL-2, IL-6, and IL-12 levels, and higher IL-10 levels than the SNI-control group (Table 1). These cytokine levels were not significantly different between *E. officinalis* extract- and naproxen-treated groups.

## 4. Discussion

In the present study, we examined the analgesic effect of *E. officinalis* extracts in PI and SNI model rats. We found that treatment of rats with *E. officinalis* extracts led to a reduction in the number of USVs in response to PI-related postoperative pain in rats, and to a decrease in hypersensitivity in response to von Frey stimulation of the hind paw, as evidenced by an increased MWT in the SNI-related neuropathic pain model rats. Preliminary analysis of the mechanism showed that *E. officinalis* extracts significantly inhibited the pain-associated pro-inflammatory cytokine levels in the DRG that were induced in response to neuropathic pain in SNI rats.

Animal models of pain play an important role in the screening and evaluation of analgesic agents. The PI model is an effective screening tool, with good reliability and predictive validity for mimicking postoperative pain in humans [22]. Incision of the plantar surface of the hind paw produced a significant reduction in MWT, as measured using the von Frey assay, and the analgesic compounds effectively reverse the incision-induced decreases in MWT for mechanical hyperalgesia [27]. As postoperative pain relief is one of the major uses of analgesics, we have previously used the PI model to test the anti-hyperalgesic effects of conventional analgesics agents, including naproxen and gabapentin [25,28]. In our current study, administration of *E. officinalis* extracts significantly attenuated mechanical hyperalgesia in response to von Frey stimulation of the injured hind paw, as evidenced by increased MWT values in the treated rats as compared to the control rats. 

The analgesic effects of *E. officinalis* extracts was also confirmed by a study of the postoperative pain-induced USV using ultrasonic microphones. As vocalization is an objective and relatively easily quantifiable measure, pain-induced USV has been examined by ultrasonic microphones, in particular USV in rats. Adult rats produce two distinct types of USVs that appear to reflect the caller’s emotional state: either a positive state (a high-pitched and short circa, ca. 50 kHz USV) or a negative state (a low-pitched and longer, ca. 22 kHz USV) [29]. The 50 kHz USV tends to be produced in non-aggressive conspecific social interactions and during play [30]. The 22–27 kHz USV have been suggested to be a measure of affective shifts in rats [31], and has been used in a variety of unconditioned models, such as those reflecting a pain- and anxiety-related status [32,33]. At 6 h or 24 h after PI, the control group emitted 22–27 kHz USV calls, which reflects pain-related behavior [34]. In our study, treatment with *E. officinalis* extracts at 300 mg/kg reduced the number of 22–27 kHz USV calls. These results, taken together, indicate that *E. officinalis* extracts may have analgesic effects on PI-related postoperative pain in rats.

The neuropathic pain animal models mimic the symptoms of chronic nerve compression in humans [35]. Chronic pain animal models are commonly defined as either nerve injury or inflammation models, but a recent study suggested that inflammatory processes are important in nerve injury-induced pain [36]. Pro-inflammatory cytokines, such as IL-6 and TNF-α facilitate neuropathic pain [37] while inhibition of pro-inflammatory cytokines or administration of anti-inflammatory cytokines, such as IL-10, reduce neuropathic pain in animal models [38]. Chronic treatment with naproxen in nerve-injured rats significantly attenuated and further delayed the development of hypersensitivity by regulating the inflammatory mediators [39]. Recently, Rijsdijk et al. reported that glucocorticoids (a group of anti-inflammatory agents) significantly reduced mechanical hyperalgesia in response to von Frey stimulation of the hind paw [40], while Li et al. reported that a corticosteroid anti-inflammatory drug, triamcinolone acetonide (TA) significantly reduced mechanical hyperalgesia in response to von Frey stimulation of the hind paw, and was highly effective at reducing spinal nerve ligation (SNL)-induced cytokine increases [41]. These reports suggest that glucocorticoids can act through trans-repression of pro-inflammatory genes to down-regulate inflammatory processes, leading to a reduced production and secretion of inflammatory cytokines, such as cyclooxygenase-2 (COX-2), IL-1β, IL-2, IL-6, IL-8, IL-12, IL-18, TNF, interferons (IFNs), and inducible nitric oxide synthase (NOS) [42,43,44]. In the present study, the *E. officinalis* extract-treated group of SNI rats showed significantly lower IL-1β, IL-2, IL-6, and IL-12 levels and higher IL-10 levels in the DRG.

The potential analgesic effects of *E. officinalis* extracts are related to its bioactive compound composition, in particular to the presence of ascorbic acid [10]. *E. officinalis* extracts are a rich source of ascorbic acid (478.56 mg/100 mL), and the levels and the presence of complex polyphenolic components are higher than those in oranges, tangerines, or lemons [45]. Previously, the anti-inflammatory or antioxidant activity of *E. officinalis* extracts have been investigated, as it contains ascorbic acid, tannins, and polyphenolic constituents [46]. Evidence from in vivo and in vitro studies have reported that *E. officinalis* extracts modulate the acute or chronic inflammatory response via antioxidant action [47,48]. Therefore, it is also hypothesized that *E. officinalis* extract may exert an analgesic effect via regulation of the inflammatory processes involved in the development of neuropathic pain.

## 5. Conclusions

In conclusion, our results demonstrated that treatment of rats with *E. officinalis* extracts significantly decreased hypersensitivity in response to von Frey stimulation of the hind paw in response to PI- and SNI-related neuropathic pain in rats. Moreover, *E. officinalis* extracts also significantly inhibited increases in the levels of pain-associated pro-inflammatory cytokines in the DRG induced by neuropathic pain in SNI rats. Although further toxicological and pharmacological investigations are needed to elucidate the detailed mechanism of action and safety, *E. officinalis* extracts could be useful in the treatment of postoperative and neuropathic pain.

## Figures and Tables

**Table 1:** Effects of *E. officinalis* extracts on cytokine expression induced by spared nerve injury (SNI) in the rat dorsal root ganglia (DRG).

Cytokines (pg/mg Protein)	SNI-Sham	SNI-Control	Naproxen	*E. officinalis*
IL-1β	1.64 ± 0.09	4.43 ± 0.34 ^a^	1.69 ± 0.28 ^b^	2.23 ± 0.27 ^b^
IL-2	7.16 ± 0.34	12.55 ± 0.72 ^a^	7.15 ± 0.23 ^b^	7.20 ± 0.33 ^b^
IL-6	48.21 ± 1.01	80.20 ± 3.57 ^a^	49.32 ± 2.66 ^b^	53.07 ± 3.03 ^b^
IL-10	6.28 ± 0.77	3.59 ± 0.17	7.36 ± 0.55 ^c^	7.52 ± 1.15 ^c^
IL-12	21.31 ± 1.11	40.56 ± 2.10 ^a^	22.05 ± 1.62 ^b^	22.31 ± 0.53 ^b^

The *E. officinalis* extract-treated group had showed significantly lower interleukin (IL)-1β, IL-2, IL-6, and IL-12 levels and higher IL-10 levels than the SNI-control group. Data represent the mean ± SEM (*n* = 9 per group). ^a^ *p* < 0.01, significant difference from the sham group. ^c^ *p* < 0.05 and ^b^ *p* < 0.01, significant difference from the SNI-control group.
